# Regulatory T Cells in the Immunodiagnosis and Outcome of Kidney Allograft Rejection

**DOI:** 10.1155/2013/852395

**Published:** 2013-06-15

**Authors:** O. Franzese, A. Mascali, A. Capria, V. Castagnola, L. Paganizza, N. Di Daniele

**Affiliations:** ^1^Division of Pharmacology, Department of Medicine of the Systems, University of Rome “Tor Vergata” Rome, Italy; ^2^Division of Nephrology, Department of Medicine of the Systems, University of Rome “Tor Vergata” Rome, Italy; ^3^Division of Internal Medicine, Department of Medicine of the Systems, University of Rome “Tor Vergata” Rome, Italy

## Abstract

Acute rejection (AR) is responsible for up to 12% of graft loss with the highest risk generally occurring during the first six months after transplantation. AR may be broadly classified into humoral as well as cellular rejection. Cellular rejection develops when donor alloantigens, presented by antigen-presenting cells (APCs) through class I or class II HLA molecules, activate the immune response against the allograft, resulting in activation of naive T cells that differentiate into subsets including cytotoxic CD8^+^ and helper CD4^+^ T cells type 1 (TH1) and TH2 cells or into cytoprotective immunoregulatory T cells (Tregs). The immune reaction directed against a renal allograft has been suggested to be characterized by two major components: a destructive one, mediated by CD4^+^ helper and CD8^+^ cytotoxic T cells, and a protective response, mediated by Tregs. The balance between these two opposite immune responses can significantly affect the graft survival. Many studies have been performed in order to define the role of Tregs either in the immunodiagnosis of transplant rejection or as predictor of the clinical outcome. However, information available from the literature shows a contradictory picture that deserves further investigation.

## 1. Introduction

Acute rejection (AR) is responsible for up to 12% of graft loss with the highest risk generally occurring during the first six months after transplantation [1]. Patient monitoring following the transplant includes physical examination, blood and urine tests, and tissue biopsy.

Rejection can often be histologically diagnosed before any variation of results obtained with laboratory tests. Many centers have introduced periodic biopsy surveillance protocols; however, to date, the clinical impact of a monitoring strategy based on biopsies is not clear [2, 3].

AR may be broadly classified into humoral and cellular rejections. In particular, antibody-mediated rejection is characterized by the presence of an antibody infiltration into the transplanted kidney, targeting HLA antigens on the peritubular and glomerular capillary endothelia, which results in complement activation, cytokine and chemokine release, and induction of adhesion molecules. This inflammatory response leads to platelet aggregation and leukocyte infiltration, which eventually contribute to the pathogenesis of acute lesions such as glomerulitis, peritubular capillaritis, microthrombi, and vessel necrosis [4].

New insights are now available into the mechanisms responsible for the immune response directed against a transplanted organ. Cellular rejection develops when donor alloantigens, presented by antigen-presenting cells (APCs) through class I or class II HLA molecules, activate the immune response against the allograft, resulting in activation of naive T cells that differentiate into subsets including cytotoxic CD8^+^ and helper CD4^+^ T cells type 1 (TH1) and TH2 cells or into cytoprotective immunoregulatory T cells (Tregs) [5]. CD4^+^ and CD8^+^ T cells infiltrate into the transplanted kidney, where they release cytokines and chemokines, causing cell death either directly or indirectly [6].

The immune reaction directed against a renal allograft has been suggested to be characterized by two major components: a destructive one mediated by CD4^+^ helper and CD8^+^ cytotoxic T cells and a protective response mediated by Tregs. The balance between these two opposite immune responses can significantly affect the graft survival [7].

Many studies have been performed in order to define the role of Tregs either in the immunodiagnosis of transplant rejection or as predictor of the clinical outcome. However, information available from the literature shows a contradictory picture that deserves further investigation. 

In this paper, we will analyze the possible role of Tregs in T-cell-mediated transplant rejection as useful biomarker for the immunological monitoring of the kidney transplantation outcome. 

## 2. Principal Mechanisms of T-Cell-Mediated AR

Transplant rejection is the consequence of the recipient's alloimmune response and consists of manifested deterioration or complete function loss of the transplanted organ. From a physiopathological point of view, AR involves both cell-mediated and antibody-mediated immunities. Both cellular and humoral responses result in the allorecognition of foreign antigens which leads to immunocompetent cell activation and the orchestration of an effector response. This process ultimately results in the damage of the transplanted organ and the graft loss, both of which can show an early or late onset, as well as a striking or gradual development.

Different cell types are involved in the graft rejection including T and B cells, macrophages, plasma cells, eosinophils, and neutrophils. T cells play a crucial role either in mounting and/or regulating alloreactive responses. The main targets of cell-mediated damage are the tubular epithelium and the endothelium. 

**Figure 1:**
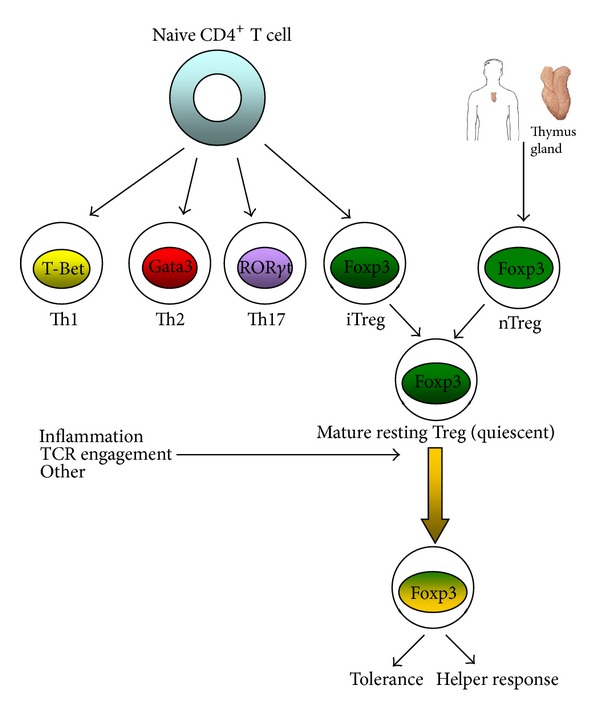
Origin and activation of Treg.

Generally acute allograft rejection starts (origins ?) when the recipient's T cells recognize the donor alloantigens presented by APCs. In particular, donor antigens are carried by immature dendritic cells from the transplanted organ to the recipient's draining lymph nodes and spleen, in a journey which induces their transformation into mature APCs [8]. After the homing of APCs to lymphoid organs, the allorecognition of foreign antigens leads to T cell activation followed by differentiation into different subpopulations (Figure 1) and the return to the graft, where they play a fundamental role in destroying the transplanted organ.

T cell infiltration into the graft is mainly at the level of postcapillary venule endothelium. Three main steps can be identified: tethering, adhesion, and transmigration [9].

Tethering consists in the attachment and subsequent rolling of T cells along the endothelium, a process mediated by endothelial selectins that not only operate as a “conveyor belt,” but can also slow down cellular movement, thus prolonging T cell interaction with the endothelium itself. This initial step is followed by T cell activation as a consequence of exposure to locally produced chemokines which induce the expression of integrins including LFA-1, thus resulting in T cell adhesion to the endothelium. In the following step of transmigration, a diapedesis-mediated T cell infiltration into the endothelial gap junctions occurs. Once T cells reach the interstitium, the induced production of metalloproteases permits the digestion of the extracellular matrix allowing T cells to move along the tissue following a chemokine-dependent gradient (chemotaxis). 

The main target of T cell activity is represented by the tubular epithelium and by the endothelium.

CD4^+^ T cells can induce cell damage either indirectly through the activation of cytotoxic CD8^+^ T cells and macrophages and/or directly through the production of inflammatory cytokines including TNF and IFN-*γ*. CD8^+^ T cells can induce damage at tubular and endothelial levels either through the release of cytotoxic molecules including perforin, granzyme B, and granulolysins or through the involvement of Fas molecule and induction of apoptosis [10]. Activated macrophages can in turn play their damaging role versus the tubular epithelium and endothelium by producing TNF-alpha and reactive oxygen species including NO. 

## 3. Treg and Tolerance

Tregs play a critical role in the maintenance of T cell homeostasis under different immune conditions. They prevent the activation of autoreactive immune responses, contribute to maintaining self-tolerance and homeostasis of the microbial flora of the gut, and promote the immunogenic escape of cancer cells [11–13]. 

### 3.1. Origin of Tregs

Tregs were identified as a CD4^+^ T cell subpopulation expressing CD25 [14] molecule and “cytotoxic T-lymphocyte antigen 4” (CTLA-4) at a similar extent to that displayed by activated T cells [15, 16]. The presence of CTLA-4 and the release of inhibitory cytokines including IL-10 and IL-35 [17, 18] suggested a suppressor phenotype for these cells and critical role in controlling the activation and function of T lymphocytes as well as of APC and NK cells. 

 Tregs originate mainly from the thymus (natural, nTregs) and from the peripheral conversion of naive CD4^+^ T cells under appropriate stimulus conditions (induced, iTregs) [19, 20]. 

Following exposure to antigens and activation of costimulatory molecules, peripheral naive CD4^+^ T cells can differentiate into different subpopulations (Figure 1): T helper 17 (Th17), Th1, Th2, and iTregs [21]. Several transcription factors contribute to the functional specialization of these subsets, including Foxp3, ROR*γ*t, T-bet, and GATA3, which activate genes involved in the control of T cell function [22–25]. 

### 3.2. Circulating Pool and Activation of Tregs

Circulating Tregs represent 5% of total lymphocytes in blood. Tregs are essential for maintaining peripheral tolerance; nevertheless, they show a quiescent phenotype when isolated from a noninflammatory environment and require functional activation for the acquisition of Treg full functional suppressive activity [26] that can be achieved following exposure to self-antigens or to antigens presented at mucosal surfaces where they can be recruited. Tregs can also be functionally activated while migrating through inflamed tissues, or by exposure to environmental conditions such as those produced by tumors [27, 28].

Inflammation plays an important role in driving the local cytokine milieu. In particular TGF-β, IL-10, and IL-2 have been shown to be critical in regulating activation and/or maintenance of the immunosuppressive functions of Tregs [29]. 

### 3.3. Regulation of Immune Responses by Tregs

Several mechanisms have been proposed to explain the role of Tregs in the control of immune responses in lymphoid and nonlymphoid tissues. Tregs produce IL-10, which is able to inhibit, either directly or indirectly, effector T cell activity during infection, autoimmunity, and cancer [30, 31]. Selective deletion of IL-10 in Tregs results in the development of spontaneous colitis and exaggerated immune responses at the skin level and lung interfaces [32] while the role of CTLA-4 has been suggested by the observation that its loss results in severe lymphoproliferative disease and spontaneous multiorgan autoimmunity [33].

Regulation of immune functions mediated by CTLA-4-expressing Tregs depends on the ability of CTLA-4 molecule to downregulate the expression of costimulatory molecules CD80 and CD86 on dendritic cells (DCs) of lymphoid tissues resulting in impaired costimulation via CD28 and defective T cell stimulation [42]. Indeed, studies have confirmed stable contacts between Tregs and DCs, confirming Treg-mediated inhibition of these cells [43, 44]. Tregs can also induce perforin-dependent cytolysis of DCs in tumour-draining lymph nodes [45]. Therefore, Tregs can control DC activity by multiple mechanisms, and this results in the inhibition of effector T cell activation and promoting of functional tolerance. 

## 4. Regulatory T Cells in the Immunodiagnosis and Outcome of Kidney Allograft Rejection

The introduction of modern immunosuppressive therapies has improved the functional prognosis of the transplanted kidney. In particular, the existing immunosuppressant drugs have been shown to decrease the progression of renal damage at 5 years towards a framework of interstitial fibrosis/tubular atrophy [46]. However, there is still much to be done in order to further decrease the percentage of graft loss. 

A current research challenge is the definition of biochemical and/or histological markers which can be considered as early signs or predictive of rejection. An ideal indicator should have the ability of discriminating between rejection and other causes of inflammation as well as to correlate with long-term prognosis and therapy efficacy.

In the search of biomarkers for the diagnosis of cell-mediated AR and prognosis of renal transplant, an increasing attention has been paid to the role of Tregs.

The role of Tregs in inducing tolerance to allogeneic grafts was demonstrated in tolerated skin allografts [47, 48]. The induction of peripheral Tregs with specificity for non-self-peptides suggested a way for obtaining antigen-specific Treg ex vivo [49]. However, although a direct and active involvement of Treg-mediated T cell suppression at the site of the tolerated transplants has been demonstrated, the specificity for donor antigens has not been fully evidenced [50]. Noteworthy, the induction of dominant allograft tolerance dependent on regulatory T cells does not necessarily result in a reduced capacity to respond to environmental pathogens [51] providing support for the development of tolerance induction protocols in clinical transplantation.

One of the most important studies dealing with the role of Tregs in renal transplantation is the work of Muthukumar et al. [34]. Urine samples from 83 kidney-transplant recipients were analyzed. Among the patients considered in the study, 36 subjects showed graft dysfunction and biopsy-confirmed AR, 29 subjects had stable allograft function and normal allograft biopsy, and 18 subjects presented allograft dysfunction and biopsies indicating chronic allograft nephropathy. The levels of Foxp3 transcripts, as a specific marker of Tregs [22], in cells obtained from urine samples of the 36 subjects with AR were higher as compared with those observed in the other 2 groups analyzed. This result contrasted with the general expectation that Foxp3 should be lower in rejection. Among the 36 episodes of AR, 26 successfully reversed, while 10 patients lost their grafts within 6 months following the acute episode of rejection. In this case a combination of Foxp3 transcripts and creatinine levels proved to be a better prognosticator of rejection reversal (90 percent sensitivity and 96 percent specificity) than Foxp3 transcripts (90 percent sensitivity and 73 percent specificity) or serum creatinine levels alone (85 percent sensitivity and 90 percent specificity). Banff histologic grade of the subjects in this case was not able to predict graft failure outcome, because there was no difference in the histological grade between the 2 groups (5 patients with IA and 5 with >IA in the group showing graft loss versus 11 patients with IA and 15 with >IA in the group with a functional graft, *P* = 0.68). Authors explained these results by suggesting that cells infiltrating the transplanted kidney would include both graft-destructive cells such as cytotoxic T cells and graft-protective Foxp3-expressing Tregs. Indeed the transient expression of Foxp3 can be a normal consequence of T cell activation without the acquisition of a Treg phenotype [52]. Consequently, graft dysfunction and response to therapy may be predicted more accurately when the heterogeneous nature of the cellular components is taken into account. 

In addition, patients who displayed both rejection and higher levels of urinary Foxp3 showed better responsiveness to steroid treatment together with significantly lower risk for graft failure as compared with subjects with lower levels of the transcription factor. 

In 2008, Aquino-Dias et al. analyzed the expression of some of the molecules mainly involved in the cytolytic attack to the graft (perforin, granzyme B, and fas-ligand), together with Foxp3 using real-time PCR from urinary cells, peripheral blood mononuclear cells, and 48 surveillance kidney biopsies from 35 patients with delayed graft functions, 20 of which showed histopathological features of AR and 28 of acute tubular necrosis [37]. All analyzed transcripts were higher in AR as compared with acute tubular necrosis. Similar results and significant correlations were observed in kidney tissue, peripheral blood leukocytes, and urinary cells for all genes analyzed. Although all correlations reached statistical significance, results concerning Foxp3 showed highest significance (94 percent sensitivity and 95 percent specificity). In a very recent study from Muthukumar et al., the urinary cell mRNA profiles for Foxp3 and other molecules where able to associate an early steroid withdrawal regimen with antithymocyte globulin induction, with excellent graft and patient outcomes in HIV-infected recipients of kidney allografts [53]. 

Mansour et al. [38] measured mRNA levels of Foxp3, Granzyme B, IFN-*γ*, IL-23, and ROR*γ*t in renal tissue obtained from 46 untreated subjects with renal allografts with borderline lymphocytic infiltrates according to Banff scheme (changes insufficient for diagnosis of AR, including foci of tubulitis with mild to moderate cortical infiltration and without intimal arteritis). Twenty-five patients were considered “nonprogressive,” as defined by serum creatinine level below 110% of baseline during the 40 days following biopsy. In contrast, 21 patients were considered “progressive,” as defined by an increase in serum creatinine level more than 110% of baseline and by repeated histologic examinations showing AR. In general, higher levels of Foxp3 mRNA were found in the nonprogressive group as compared with those observed in the progressive group.

In a retrospective study, Xu et al. [39] analysed 125 surveillance biopsies displaying interstitial T-lymphocyte infiltration between nonatrophic tubules in the cortex, 14 with subclinical rejection, 32 with borderline change, and 79 showing interstitial T-lymphocyte infiltration without obvious pathological abnormalities according to Banff criteria. 

All previously described cases were classified into two groups: a regulatory phenotype (RP) group, characterized by Foxp3^+^-infiltrating T lymphocytes in biopsies, and a cytotoxic phenotype (CP) group, which was dominated by Granzyme B^+^-T lymphocytes. No patient of the RP group developed any AR during nearly 5 years of followup, while subjects of the CP group developed biopsy-proven or clinical diagnostic AR within 1 year after biopsy.

The clinical significance of the ratio between IL-17-secreting cells and Treg infiltration in renal allograft tissues with acute T-cell-mediated rejection (ATCMR) was investigated by Chung et al. [40] on 56 patients with biopsy-proven ATCMR, who were divided into the Foxp3-high group (with Log Foxp3/IL-17 > 0.45) and the IL-17-high group (with Log Foxp3/IL-17 < 0.45).

The IL-17-high group showed an allograft function significantly decreased as compared with that displayed by the Foxp3-high group, together with higher severity of interstitial and tubular injuries and lower 1-year (54% versus 90%, *P* < 0.05) and 5-year (38% versus 85%, *P* < 0.05) allograft survival rates. Multivariate analysis revealed that the Foxp3/IL-17 ratio was a significant predictor for allograft outcome. 

 The level of circulating Tregs at peripheral blood level and the association with long-term graft survival were analyzed by flow cytometry in 90 kidney transplant recipients [41]. Patients who maintained high Treg levels (above 70%) at both 6 and 12 months displayed a better long-term graft survival at 4 and 5 years followup. 

The previously mentioned study would suggest that Tregs may play a role in antagonizing the inflammatory state associated with kidney transplantation and may possibly be considered as a prognostic factor of outcome. However, several studies show divergent data potentially contrasting with this vision.

Veronese et al. [35] analyzed 73 renal transplant biopsies selected for the diagnosis of acute cellular rejection (ACR) type I or type II, acute humoral rejection (AHR), or calcineurin inhibitor toxicity (CNI). The number of Tregs was found to be significantly higher in ACR type I and type II, as compared with that observed in AHR and CNI toxicity; 96% Foxp3^+^ cells were CD4^+^ T lymphocytes aggregated within renal tubules. However, Kaplan-Meier analysis of 2-year graft survival in patients with ACR type I or type II showed a lower survival rate in patients with higher Foxp3 scores as compared with the other group.

Bunnag et al. [36] analyzed Foxp3 mRNA expression in 83 renal transplant biopsies for causes linked to histopathology. Kidneys with T-cell-mediated rejection, antibody-mediated rejection, and mixed rejection showed higher Foxp3 expression as compared with kidneys without rejection. According to Banff classification, higher Foxp3 expression was associated with higher levels of interstitial inflammation and tubulitis. In their multivariate analysis, CD4 positivity, and not Foxp3 mRNA expression, was independently associated with graft survival.

Batsford et al. [54] found no association between Foxp3 T cell expression and graft function one year after transplantation. However, this study was affected by the reduced number of samples and the choice of excluding patients with a degree of rejection higher than type 1 TCMR. 

Dummer et al. [55] have recently observed that intragraft expression of both Foxp3 mRNA and protein was not associated with a better allograft outcome, analysed in terms of graft function and survival at 5 years after transplantation in 96 kidney transplants.

## 5. Concluding Remarks

The analysis of some of the most important recent studies dealing with Tregs used as possible biomarker of acute kidney transplant rejection and/or prognostic factor related to the graft survival (see Table 1) inspires some critical observations. 

First of all it must be pointed out that most of the times the final assessment of the studies is affected by the modest statistical validity of the analyzed sample due to the small number of patients included.

The consideration that analysis of Foxp3 mRNA and Foxp3^+^ CD4^+^ T cells is often performed on bioptic samples is a critical element to take into account. The use of a graft survival biomarker could be able to improve the prognostic validity of the procedure, also in terms of evaluation of response to immunosuppressive therapies. On the other hand, it must be considered that renal biopsying for the diagnosing of allograft rejection is an invasive and time-consuming procedure with some risk of complications and not easily manageable for all patients. Therefore, in the general followup of transplanted patients, a noninvasive test could be more advantageous, although this option still needs to be confirmed on additional cohorts taking into account all the aspects considered earlier. 

Lastly, the different immunosuppressive therapies employed in the available studies and the potential effects on Treg expression and function constitute another critical variable to take into account in the evaluation of Treg function in the allograft outcome. Recent clinical studies have demonstrated how different immunosuppressive drugs can influence differently the number and function of Tregs, by inducing stimulation, inhibition, or even noninterference. In particular, while corticosteroids and rapamycin have been shown to improve the suppressive activity and survival of Tregs, other treatments as calcineurin inhibitors (CIs) have been shown to affect Treg function [56]. However, it is difficult to differentiate the effects of different immunosuppressants, although the use of selective pharmacological treatments able to regulate the suppressive function of Tregs would be attractive in organ transplantation.

## Figures and Tables

**Table 1:** Characteristics of the described studies.

Study	Type of patient	Patients* (*n*°)	Treg identification	Treg or Foxp3 expression	Characteristic
Muthukumar et al. (2005) [34]	KT with AR	36	Foxp3 mRNA in urine	Elevated	Survival rate graft
Veronese et al. (2007) [35]	KT with AR	59	Foxp3 CD4^+^ in kt	Elevated	Survival rate graft
Bunnag et al. (2008) [36]	KT with AR	31	Foxp3 mRNA in kt	Elevated	Survival rate graft
Aquino-Dias et al. (2008) [37]	KT with AR	20	Foxp3 mRNA in kt, PBL, urine	Elevated	Diagnosis of AR
Mansour et al. (2008) [38]	KT with BL changes	46	Foxp3 mRNA in kt	Reduced in PG	Outcome of BL changes
Xu et al. (2012) [39]	KT	125	Foxp3-positive T lymphocytes in kt	Elevated in RPG	Outcome of graft
Chung et al. (2012) [40]	KT with AR	56	Foxp3/IL-17 secreting cells ratio in kt	Elevated (in SRGa)	Survival rate graft
San Segundo et al. (2012) [41]	KT	90	Foxp3 CD4^+^ CD25^+^ in PBL	Elevated (in SRGa)	Survival rate graft

*The number of patients does not refer to the total number of patients in each study, but to the subpopulation considered.

KT: kidney transplantation; AR: acute rejection; kt: kidney tissue; PBL: peripheral blood lymphocytes; BL changes: borderline changes; PG: progressive group; RPG: regulatory phenotype group; SRGa: patients with augmented survival rate graft.
